# The Effective and Safe Way to Use Crusade Microcatheter-Facilitated Reverse Wire Technique to Solve Bifurcated Lesions with Markedly Angulated Target Vessel

**DOI:** 10.1155/2019/2579526

**Published:** 2019-04-11

**Authors:** Jingang Cui, Xiaowei Jiang, Shubin Qiao, Lijian Gao, Jiansong Yuan, Fenghuan Hu, Weixian Yang, Runlin Gao

**Affiliations:** Cardiology Department, State Key Laboratory of Cardiovascular Disease, Fuwai Hospital, National Center for Cardiovascular Diseases, Chinese Academy of Medical Sciences and Peking Union Medical College, Beijing, China

## Abstract

**Objectives:**

We aim to present a new way to introduce reverse wire in crusade microcatheter-facilitated reverse wire technique to solve markedly angulated bifurcated lesions.

**Background:**

Markedly angulated coronary bifurcation lesions are still one of the considerable challenges of treatment with percutaneous coronary intervention especially with severe proximal stenosis. Microcatheter-facilitated reverse wire technique improved the efficacy of crossing a guide wire to such an extremely angulated complex targeted vessel. However, there has been a debate regarding what kind of curve was the best to introduce reverse guide wire in this technique.

**Methods:**

We analyzed 7 patients who were admitted to Fuwai Hospital and underwent antegrade wiring which failed. Crusade microcatheter-facilitated reverse wire technique with simple short tip one round curve was used successfully to solve in all 7 bifurcation lesions. We investigated the bifurcation lesion's characteristics and details of the reverse wire technique procedures.

**Results:**

All 7 bifurcations exhibit both a smaller take-off angle and a larger carina angle and severe proximal significant stenosis. After having suitable size of balloon predilation, reverse wire with simple short distal one curve was delivered to distal segment of targeted vessel successfully. We performed all PCI procedures without any complications and no major adverse cardiac event was observed during hospitalization.

**Conclusions:**

In solving markedly angulated bifurcated lesions, especially with severe proximal stenosis, crusade microcatheter-facilitated reverse wire technique with simple short tip one curve is an effective and safe way of wiring.

## 1. Introduction

Safe guidewire placement in the main vessel (MV) and in the side branch (SB) is necessary for successful percutaneous coronary interventions (PCI) in bifurcated lesions. Bifurcated lesion with small take-off angel is an important predictive factor, and targeted vessel wiring could be particularly difficult.

The reversed guide wire technique, firstly reported by Kawasaki et al. in 2008, has been improved with utilizing the support of dual lumen catheter and is a very useful guide wire manipulation technique to cross a guide wire to such an extremely angulated complex targeted vessel. In reverse wire technique, two curves are created at the guide wire tip, and first curve is a short tip one and secondary curve is an opposite longer proximal one. However, especially in markedly angulated bifurcated lesions with tight proximal stenosis, there had been different opinions about how to shape the secondary curve: sharply [1] (easier to pass the proximal stenosis segment) or roundly [2] (easier to delivery device through reverse wire).

We have experienced 7 cases of difficult vessel access with severe proximal stenosis. The microcatheter-facilitated reverse wire technique without the secondary curve was successful to cross a guide wire to extremely angulated complex targeted vessel. Therefore, the present study aimed to discuss what kind of curve in reverse wire is more effective in markedly angulated bifurcation. 

## 2. Materials and Methods

### 2.1. Study Design and Patient Population

This is a retrospective study conducted at Fuwai Hospital. We enrolled 7 patients in whom conventional antegrade wiring to branch vessel was easily done, but wiring to target vessel had failed, and in whom a reverse wire technique was used in an attempt to approach target vessels of bifurcation lesions with a small take-off angel. This study was approved by the Ethics Committee of Fuwai Hospital. All participants provided their written informed consent to agree with using their clinical information.

Quantitative assessments were performed using the Cardiovascular Measurement System (QAngio XA 7.2, MEDIS), a personal computer-based system, for offline quantitative angiographic analysis. The take-off angel indicates the angle between the proximal untargeted vessel and targeted vessel, and the carina angle indicates the angel between the distal untargeted vessel and the targeted vessel.

### 2.2. Preparation of the Reverse Wire System

**Figure 1:**
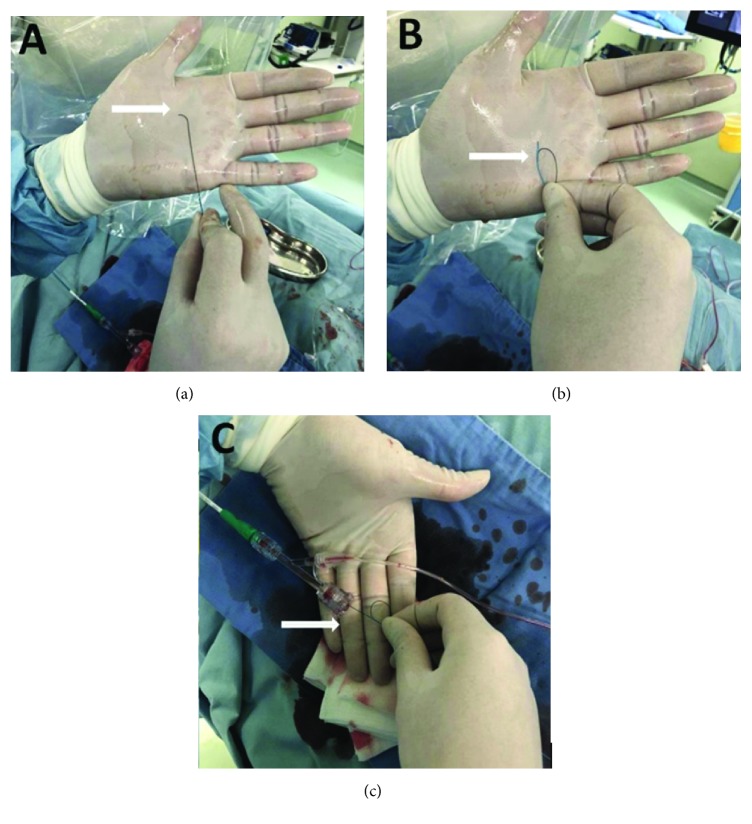
Microcatheter-facilitated reverse wire technique. (a) A short tip curve (as indicated by white arrow) which fits the diameter of the untargeted vessel in a retrograde. (b) Softly folding the guide wire (as indicated by white arrow), in opposite direction of the first curve at about 3 cm away from the wire tip approach. (c) The reverse wire system was inserted through the hemostatic valve into guiding catheter (as indicated by white arrow).

A crusade catheter and fielder FC (Asahi Intecc) guide wire were used exclusively for the reverse wire technique in all 7 patients. In aspect of guide wire shape, our method of wire shape had many differences from past studies [1–3]. The guide wire was introduced into the over-the-wire lumen of the crusade catheter and shaped by a tip curve (0.2-0.5 cm) that fit the diameter of the untargeted vessel and without being followed by a secondary shape curve. Then, a bend of guide wire is fixed softly with the crusade catheter in the opposite direction of the tip curve at 3 cm away from the tip of the guide wire in a retrograde approach (Figure 1).

### 2.3. Details of the Procedure (Figure 2)

The first guide wire was placed in the untargeted vessel. Before the insertion of a guide wire into the untargeted vessel with a marked angulation, due to the tight stenosis proximal bifurcation, we usually inflate a suitable size of balloon catheter to modify the plaque distribution of untargeted vessel and make reverse wire system pass the stenosis proximal easily.

The first guide wire in the untargeted vessel supports delivery of the crusade catheter alone with the bent wire down to the untargeted vessel with the tip of the reversely bent wire remaining distal to the carina of the targeted vessel.

After pulling the crusade microcatheter back to the parent vessel, the reversely bent wire was pulled back slowly and manipulated gently so that the wire tip directs into the ostium of the target vessel. Some rotational guide wire is required to deliver the wire down to the distal segment of target vessel. Using the balloon trapping method, the crusade catheter is removed.

### 2.4. Statistical Analysis

Descriptive analysis was performed. Results are presented as percentage for categorical variable, ranging from minimum value to maximum value for continuous variables.

## 3. Results

We demonstrated the lesion's characteristics and procedural outcomes and the details of the PCI procedures by analyzing the records and angiography films of the PCI.

### 3.1. Baseline Demographics and Bifurcation Lesions Characteristics

As displayed in Table 1, there are 7 cases in which conventional antegrade wiring failed after being used, and the carina angles of the targeted vessel ranged from 125 to 160 degrees. All patients required the reverse wire technique to access target vessel of the bifurcation lesion due to an acutely angulated carina. Most of the patients were male 6 (85.7%), the age range was from 43 to 80 years old, and all of the patients presented with angina 7 (100%). The bifurcation lesions were located in left anterior descending and diagonal coronary arteries 6 (85.7%), or LAD and LCX 1 (14.3%). The type of bifurcation according to the Medina classification [4] was 1,1,1 4 (57.1%) or 1,1,0 3 (42.9%). The targeted vessel was the LAD in 7 (100%) patients. 3 (42.9%) lesions were chronic total occlusion lesions and 4 (57.1%) severe stenosis lesions involving the parent vessels proximal to the bifurcation lesion.

### 3.2. PCI Procedural Characteristics

**Figure 3:**
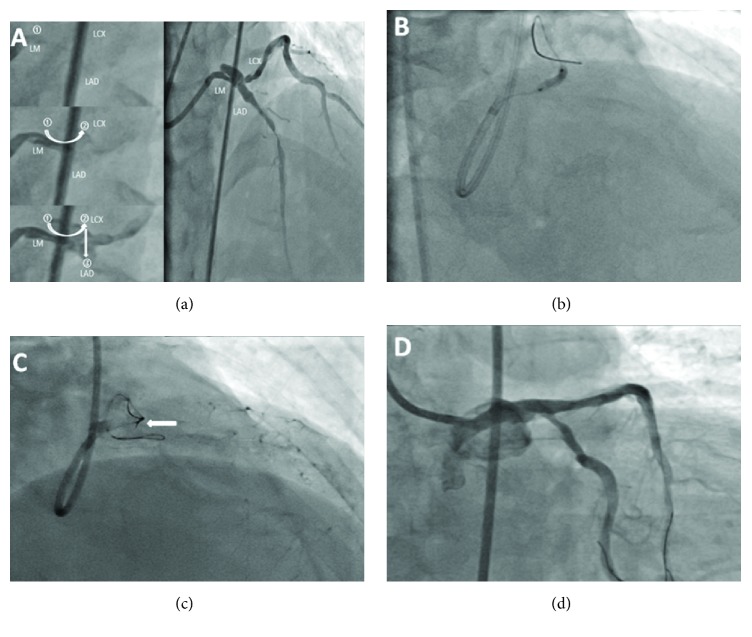
LM bifurcations with markedly angulated LAD vessel and severe proximal stenosis. (a) Direction of blood flow was marked with Arabic numerals and white arrow, and the order contrast appeared was LM, LCX, to LAD. (b) The balloon (2.5mm in diameter) predilation in LM-LCX after wring first guide wire in the LCX vessel. (c) The reverse wire tip (as white arrow) directs into the ostium of the LAD vessel. (d) Angiographic image obtained after the PCI.

**Figure 4:**
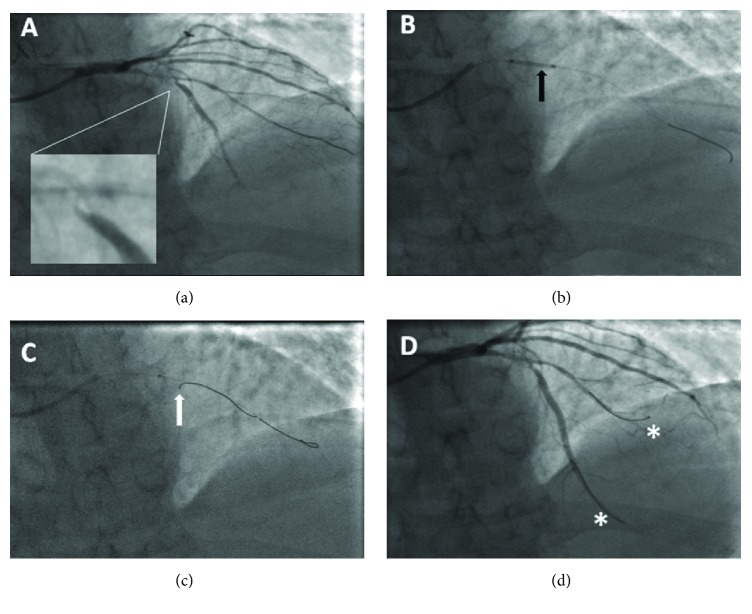
LAD bifurcations. (a) Markedly angulated LAD and proximal chronic total occlusion. (b) The balloon (1.5mm in diameter) predilation in LAD-D after wring first guide wire in the diagonal vessel. (c) The reverse wire tip (as white arrow) direct into the ostium of the LAD vessel. (d) Angiographic image obtained after reverse wire technique successful (as white asterisk).

As described in Table 2, there were 5 cases (71.4%) approached via the radial artery and 2 cases (28.6%) via the femoral artery. A 6 Fr guiding catheter was used in 5 cases (71.4%), and a 7 Fr guiding catheter was used in 2 cases (28.6%). Patients with severe stenosis (Figure 3) or CTO (Figure 4) of the parent vessel required balloon predilatation to allow passage of the crusade catheter alone with the reversely bent wire after the first wire arrived at distal segment of untargeted vessel. The diameter of predilation balloon (1.5-2.5mm) used was as small as possible under the condition of passage of the reverse system. No cases of vascular dissection and occlusion were noted.

For the cases with successful wiring, successful revascularization was achieved in 5 patients using the single stent technique (71.4%), 1 patient using the two stents technique (14.3%), and 1 patient using a drug-eluting balloon (14.3%), resulting in a procedural success rate of 100%. No cases of vascular perforation or dissection, thromboembolism, or other remarkable events were noted during the interventions.

## 4. Discussion

The complex pattern of bifurcation coronary anatomies and the different pattern of atherosclerotic disease distribution may render targeted vessel wiring highly challenging. There are several techniques which have been proposed to overcome the difficulty of guide wire crossing in markedly angulated bifurcated lesions [5]. The balloon occlusion technique in the target vessel can sometimes allow a much easier introduction of the guide wire to the SB. The venture wire-control catheter is the only deflectable catheter for guidewire delivery in this challenging anatomy [6]. However, severe stenosis with a large plaque burden in the proxima vessel is angiographic predictors of difficult targeted vessel wiring [7].

The reversed guide wire technique, firstly reported by Kawasaki et al. in 2008[3], is currently performed with some modifications. And the main differences from the original technique include the facilitation of a microcatheter and the different bending point. Microcatheter-facilitated reverse wire technique includes two shape curves: the first is a short distal and the second is an opposite proximal. To deal with bifurcated lesions with markedly angulated and severe proximal stenosis, there remains no consensus about the second shape curve: “sharp” or “round” using crusade microcatheter-facilitated reverse wire technique, owing to sharp curve with advantage of being easier to pass the proximal stenosis segment and round curve being easier to deliver device through reverse wire.

Most cases exhibit significant stenosis proximal to the bifurcation (100% in our study), which often hampers the delivery of the reverse wire system. Because the sharp curved reverse wire system is easier to pass the stenosis as compared to the roundly curved system, a sharp curve was recommended in some study [1]. In our study, even when a sharp curve was employed, we could not deliver the reverse wire system beyond the legion of severe stenosis located just proximal to the bifurcation. To overcome this problem, before the insertion of a guide wire into the untargeted vessel, a suitable size of balloon predilation to modify the plaque distribution of nontargeted vessel is required, making reverse wire system passes the stenosis proximal easily.

Also past studies have demonstrated that balloon predilation of stenotic lesions before the insertion of a guide wire into the targeted vessel with a marked angulation may help this phenomenon but carry a risk of causing a plaque or carina shift, resulting in targeted vessel occlusion [8]. In our study, the targeted vessel was target vessel, and the untargeted vessel was branch vessel; the diameter of target vessel was larger than untargeted vessel. TIMI flow of targeted vessel was not influenced and without any vascular dissection after having suitable size of balloon dilation. There are two causes which could be attributed to this phenomenon. First, the diameter of predilation balloon is of small size, and we dilated the severely stenotic legion proximal to the bifurcation with semicompliant balloon for the purposes of lesion modification, not leading to occlusion of targeted vessel, also being able to successfully deliver the system into the untargeted vessel without causing any vascular injury or other remarkable complications. Second, the plaque distribution in target vessel was in opposite direction of untargeted vessel (in Figure 3(a)), and the diameter of targeted vessel was large enough to accommodate the modification of plaque after predilation in untargeted vessel. On the contrary, if the targeted vessel is branch vessel, and the untargeted vessel is main vessel, because of the shift of plaque and relatively small diameter of branch vessel, the predilation in target vessel may result in the occlusion of branch vessel. We supposed that it is safe to predilate in branch vessel before target vessel wiring.

We did not shape the second curve after the short tip curve, and once the wire tip curve directs into the ostium of the target vessel, with the help of wire tension to restore the original line shape, we could deliver the wire down to the targeted vessel distal segment smoothly and easily. Watanabe et al. [2] demonstrated that compared with the sharp, the round curved guide wire is definitely advantageous for delivery after reverse wiring. But they did not compare the differences between with secondary curve and without secondary curve in the reverse guide wire. In our study, the simple one tip curved reverse wire, without secondary shaped curve, kept the tension of restoring the original line shape, making it more easy to deliver guide wire to distal of targeted vessel before the tip of reverse wire engages with the ostium of targeted vessel during the wire pullback. This advantage would remain until the bend of reverse wire passes the location of bifurcation. These findings indicate that modification on reverse wire technique is effective and safe in overcoming challenging wiring for highly angulated targeted vessel with severe proximal stenosis.

*Limitation*. This study has some limitations. First, this is a retrospective observational analysis of a small number of patients without control group. Second, the majority of procedures were performed by an experienced operator, and the method may not be applicable for less experienced operators. At last, the bifurcation lesions in our study only included LAD and diagonal coronary arteries, LAD and LCX, but not right coronary artery. However, we also used simple short tip one curve to perform PCI easily and successfully in bifurcated lesions with markedly angulated obtuse marginal vessel, and without the help of predilation without proximal stenosis. We speculated that simple short tip one curve of reverse wire also could be extendedly used in other coronary artery bifurcated lesions with markedly angulated target vessel, with or without severe proximal stenosis.

## 5. Conclusion

In solving markedly angulated bifurcated lesions, especially with severe proximal stenosis, crusade microcatheter-facilitated reverse wire technique with simple short tip one curve is an effective and safe way of wiring.

## Figures and Tables

**Figure 2:**
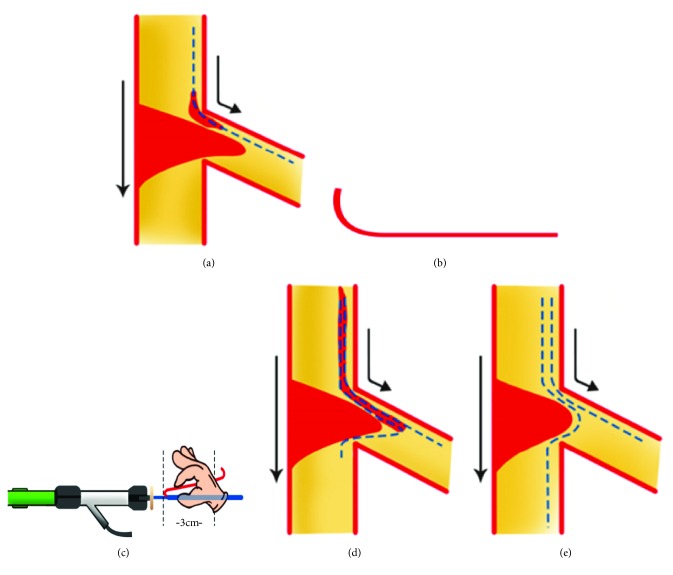
Diagrammatic representation of the microcatheter-facilitated reverse wire technique. (a) Due to the tight stenosis proximal bifurcation, we usually inflate a suitable size of balloon catheter to modify the plaque distribution of untargeted vessel and make reverse wire system pass the stenosis proximal easily. (b) A short tip curve was made on guidewire. (c) Softly folding the guide wire with a smooth round curve and inserting it into the hemostatic valve. (d) After pulling back the reverse system proximal to the bifurcation lesion and some rotations, the reverse wire tip directs into the ostium of the target vessel. (e) Deliver the reverse wire down to the distal segment of target vessel.

**Table 1:** Baseline demographics and bifurcation lesions characteristics.

Case number	Sex	Age (years)	Untargeted vessel	Targeted vessel; TIMI flow	Proximal stenosis (%)	Medina classification	Take-off angle (degrees)	Carina angle (degrees)
1	Male	50	D	LAD; II	100	1,1,1	30	150
2	Female	57	LCX	LAD; III	90	1,1,0	55	125
3	Male	43	D	LAD; II	100	1,1,1	45	135
4	Male	66	D	LAD; II	100	1,1,1	45	135
5	Male	68	D	LAD; III	90	1,1,1	25	155
6	Male	54	D	LAD; II	95	1,1,0	50	130
7	Male	80	D	LAD; III	90	1,1,0	20	160

D, diagonal branch; LAD, left anterior descending; LCX, left circumflex.

**Table 2:** PCI procedural characteristics.

Cases number	Approach	Guiding catheter (Fr)	Guide wire	Microcatheter	Balloon predilation (mm*∗*mm); location	TIMI flow in LAD after predilation	Stenting strategy	Procedure success
1	Radial	6	Fielder FC	Crusade	1.5*∗*15; LAD-D	III	Single	Yes
2	Femoral	7	Fielder FC	Crusade	2.5*∗*15; LM-LCX	III	Single	Yes
3	Radial	6	Fielder FC	Crusade	1.5*∗*15; LAD-D	III	Single	Yes
4	Femoral	7	Fielder FC	Crusade	1.5*∗*15; LAD-D	III	Crush	Yes
5	Radial	6	Fielder FC	Crusade	2.5*∗*15; LAD-D	III	Single	Yes
6	Radial	6	Fielder FC	Crusade	2.0*∗*15; LAD-D	III	Single	Yes
7	Radial	6	Fielder FC	Crusade	2.0*∗*15; LAD-D	III	DEB	Yes

TIMI, thrombolysis in myocardial infarction; DEB, drug eluting balloon.

## Data Availability

The data used to support the findings of this study are available from the corresponding author upon request.
